# Supplementary figures and images for: γδ T cells respond directly and selectively to the skin commensal yeast Malassezia for IL-17-dependent fungal control
Source: PLoS Pathog. 2024 Jan 12;20(1):e1011668. doi: 10.1371/journal.ppat.1011668 (PMC10810444; doi:10.1371/journal.ppat.1011668)

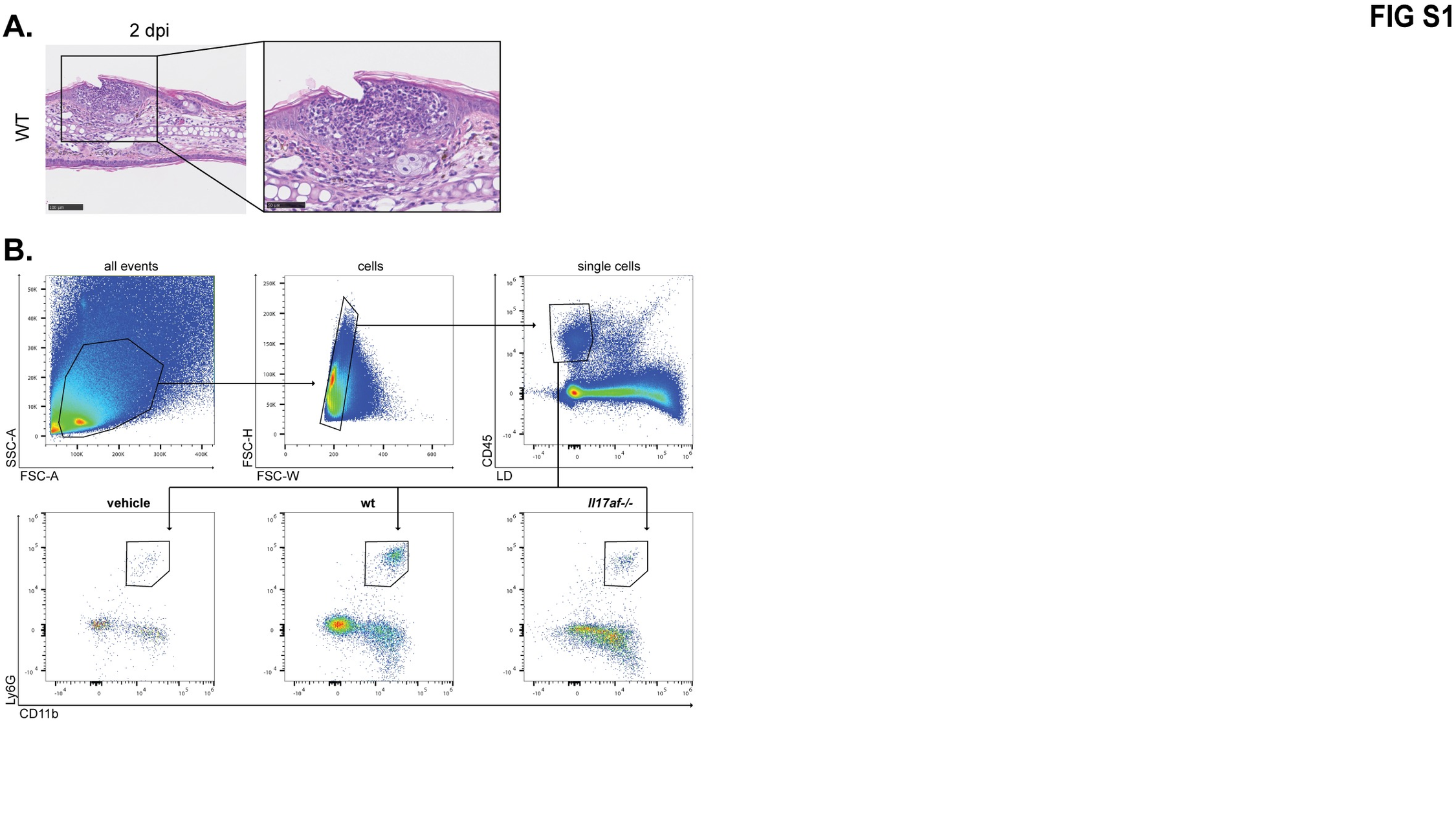

Supplement: S1 Fig — The ear skin of wild type (WT) and Il17af-/- mice was associated with M. pachydermatis or treated with olive oil (vehicle control). A. Hematoxylin and eosin-stained WT ear tissue section on day 2 after M. pachydermatis association shown in Fig 1C and magnification of the indicated area. B. Gating strategy for neutrophils (Ly6Ghi CD11b+) among viable CD45+ cells in the ear skin of WT and Il17af-/⁻ mice 4 days after association with M. pachydermatis. (TIF) [file ppat.1011668.s001.tif]

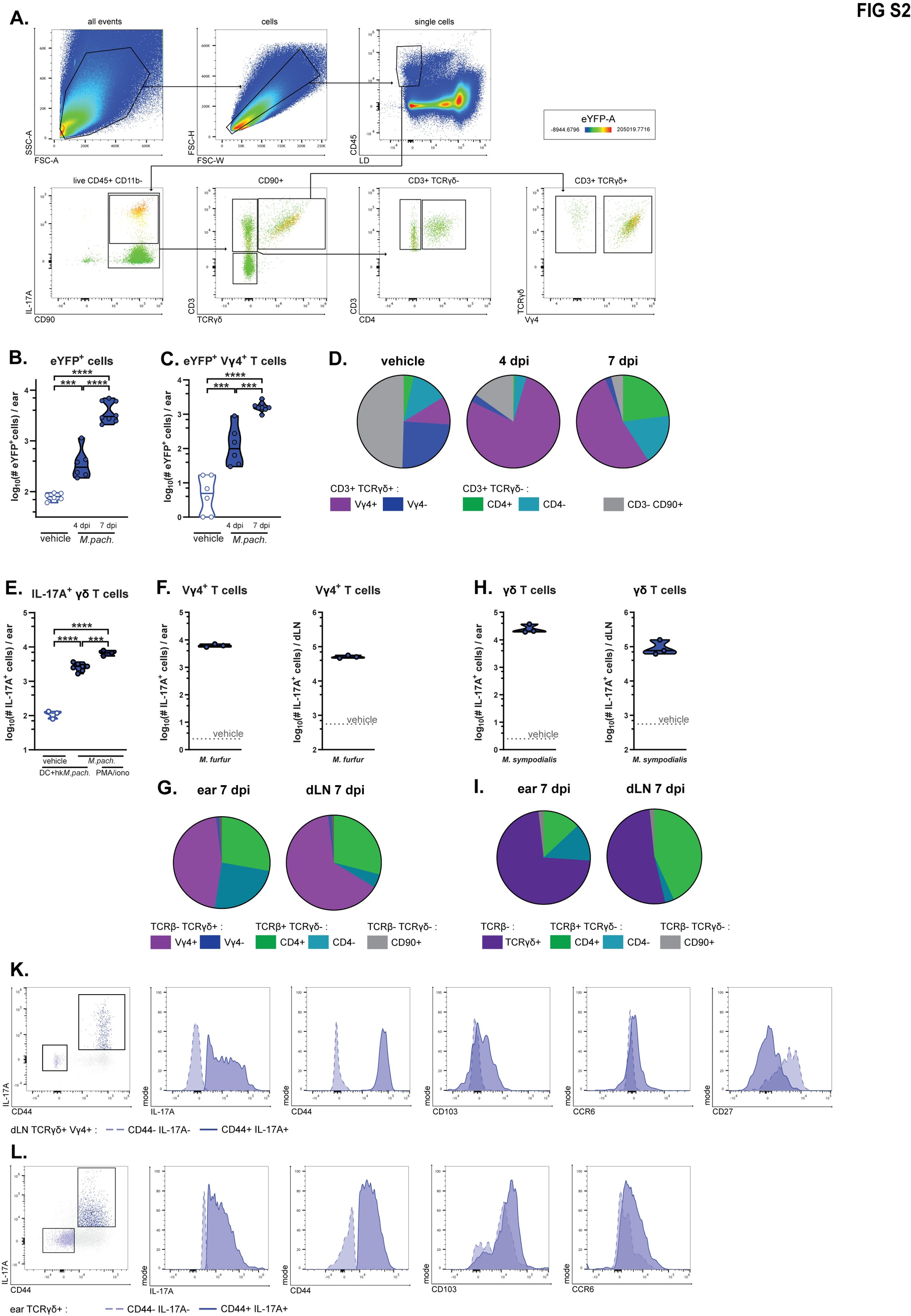

Supplement: S2 Fig — A-D. The ear skin of ll17aCre R26ReYFP fate reporter mice was associated with M. pachydermatis and the IL-17-expressing cellular subsets were assessed by flow cytometry analysis of eYFP reporter expression. A. Gating strategy for identifying the eYFP+ cellular subsets among viable CD45+ CD11b⁻ single cells. eYFP expression levels are indicated by a colour scale. B.-C. Quantification of eYFP+ cells among overall skin CD90+ cells (B) or Vγ4+ TCRγδ+ CD90+ T cells (C) at the indicated time points (dpi) and in uninfected control animals (vehicle). D. Proportion of eYFP+ cells among the indicated cell populations in the ear skin at the indicated time points (dpi) and in uninfected control animals (vehicle). Data in A—D are from two independent experiments per time point with 2–4 mice per group; each symbol represents one mouse; the median of each group is indicated. E. IL-17A+ cells after the indicated re-stimulation in M. pachydermatis-associated or vehicle control mice on day 7. Data are pooled from two independent experiments with 3–4 mice per group. Each data point represents one mouse, the median is indicated. F.-I. WT mice were associated with M. furfur (F, G) or M. sympodialis (H, I) for 7 days and γδ T cells were quantified in ear skin and dLN. Quantification of IL-17A+ cells among Vγ4+ TCRγδ+ CD90+ T cells (F) or among TCRγδ+ CD90+ T cells (H) after ex vivo re-stimulation with PMA and ionomycin (skin) or with heat-killed Malassezia spp.-pulsed DCs (dLN). Proportion of IL-17A+ cells among the indicated cell populations in the ear skin and dLN (G, I). Data in F-I are from one experiment with 3 mice per group. K.-L. Phenotype of IL-17A+ Vγ4+ (dark blue, solid line) and IL-17A- Vγ4+ γδ T cells (light blue, dashed line) in dLN (K) and ear skin (L) after restimulation with hkM.pach.-pulsed DCs or PMA/ionomycin, respectively. Histograms are from three concatenated samples. Data are from three (K) or two (L) independent experiments with 3–5 mice per group. Statistical si [file ppat.1011668.s002.tif]

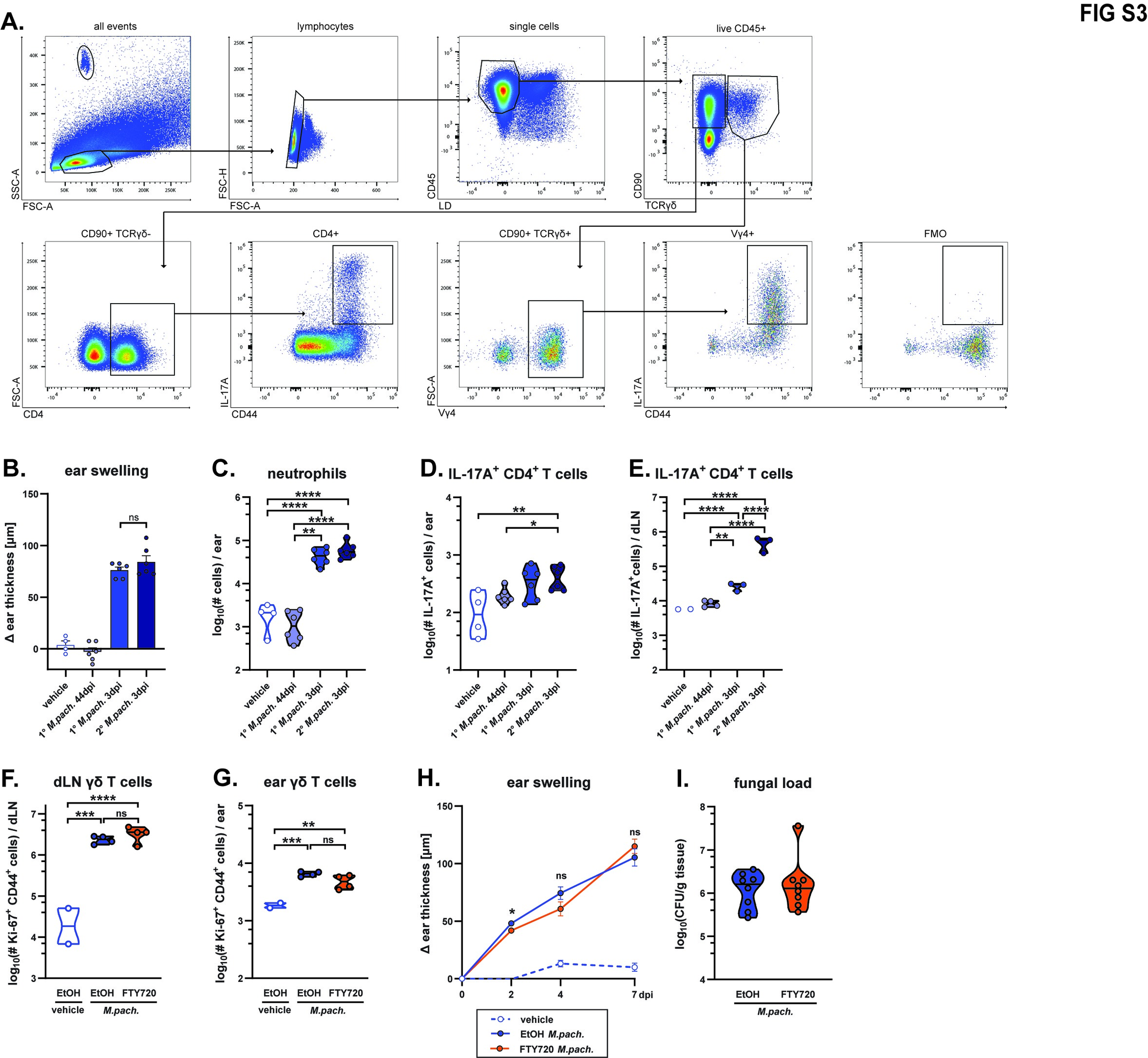

Supplement: S3 Fig — A-E. WT mice were associated with M. pachydermatis (M.pach.) once or twice or treated with olive oil (vehicle control) as in Fig 4A–4E. Gating strategy for quantifying IL-17 producing TCRγδ- CD4+ T cells and Vγ4+ TCRγδ+ T cells (A). Increase in ear thickness (B). Total numbers of neutrophils (C) and TCRγδ- IL-17+ CD4+ T cells in the skin (D). Total numbers of M. pachydermatis-reactive IL-17A+ TCRγδ- CD4+ T cells in dLN (E). Data in B-D are compiled from two independent experiments, and in E are from one representative of two independent experiments with 2–4 mice per group. Each symbol represents one mouse. The mean+SEM per group is indicated in B, the median per group is indicated in C-E. F.-I. WT mice were associated with M. pachydermatis and treated with FTY720 or EtOH solvent control as in Fig 4F–4I, but analyzed at 4 dpi instead of 7 dpi. Total numbers of Ki-67+ CD44+ γδ T cells in dLN (F) and ear skin (G), increase in ear thickness (H), and skin fungal load (I). Data are from one representative of two (F-G) or pooled from two (H-I) independent experiments. Each symbol represents one mouse; the median per group is indicated. Statistical significance was determined using one-way ANOVA (B-G), two-way ANOVA (H), or Student’s t test (I). *p<0.05, **p<0.01, ***p<0.001, ****p<0.0001. (TIF) [file ppat.1011668.s003.tif]

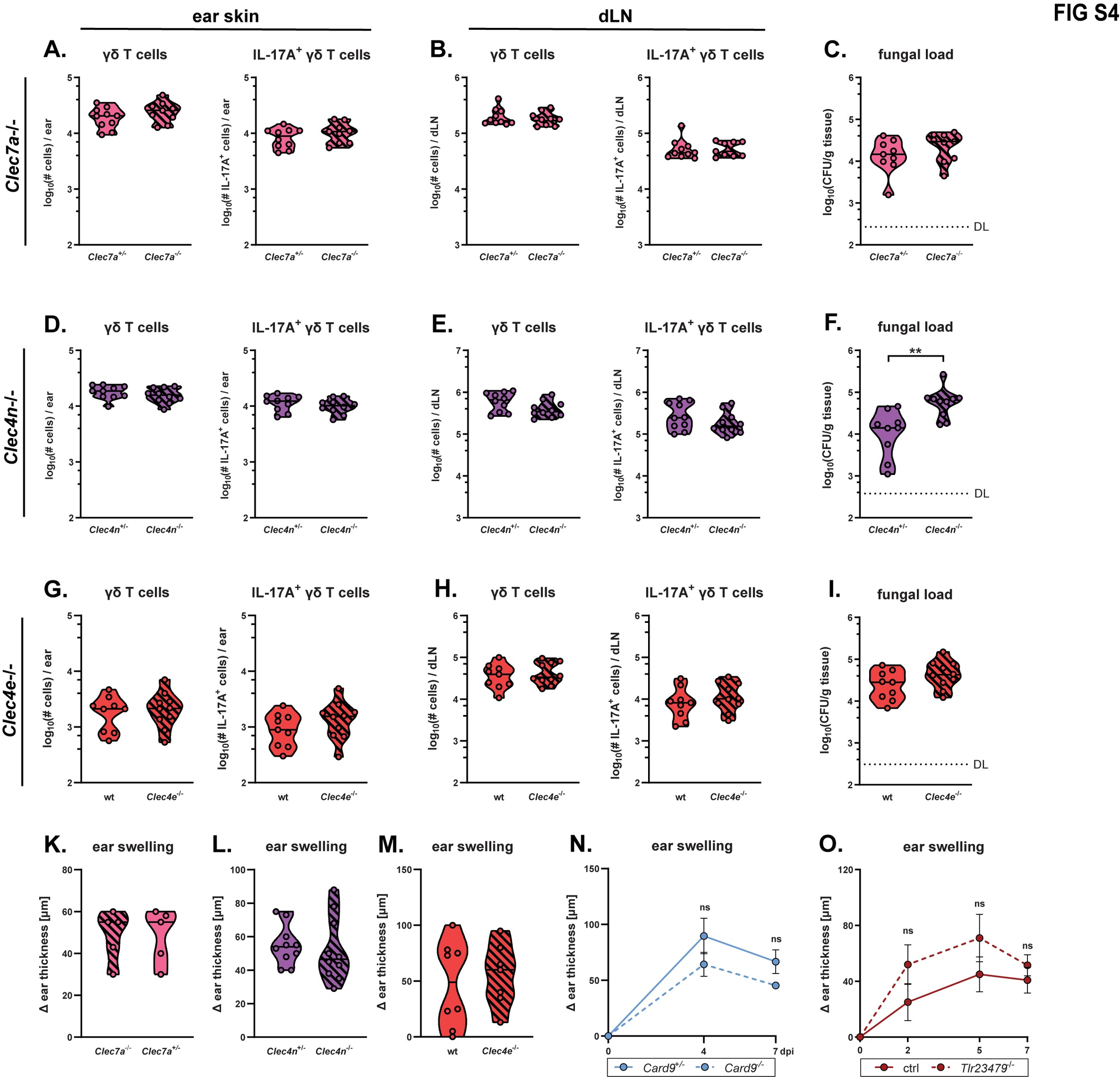

Supplement: S4 Fig — The ear skin of Clec7a-/- and Clec7a+/- littermate control mice (A-C, K), Clec4n-/- and Clec4n+/- littermate control mice (D-F, L), irradiation chimeras reconstituted with Clec4e-/- or WT control bone marrow (G-I, M), Card9-/- and Card9+/- littermate control mice (N) or Tlr23479-/- and control mice (O) was associated with M. pachydermatis. After 7 days, γδ T cells in skin and dLN were quantified (A, D, G) and analyzed for IL-17 production (B, E, H). Skin fungal load (CFU) (C, F, I). Increase in skin ear thickness (K-O). Data are pooled from two (A-F, K, L, O) or three (G-I, M, N) independent experiments with 3–5 mice per group. Each symbol represents one mouse. The median of each group is indicated. DL, detection limit. Statistical significance was determined using unpaired Student’s t-test. **p<0.01. (TIF) [file ppat.1011668.s004.tif]

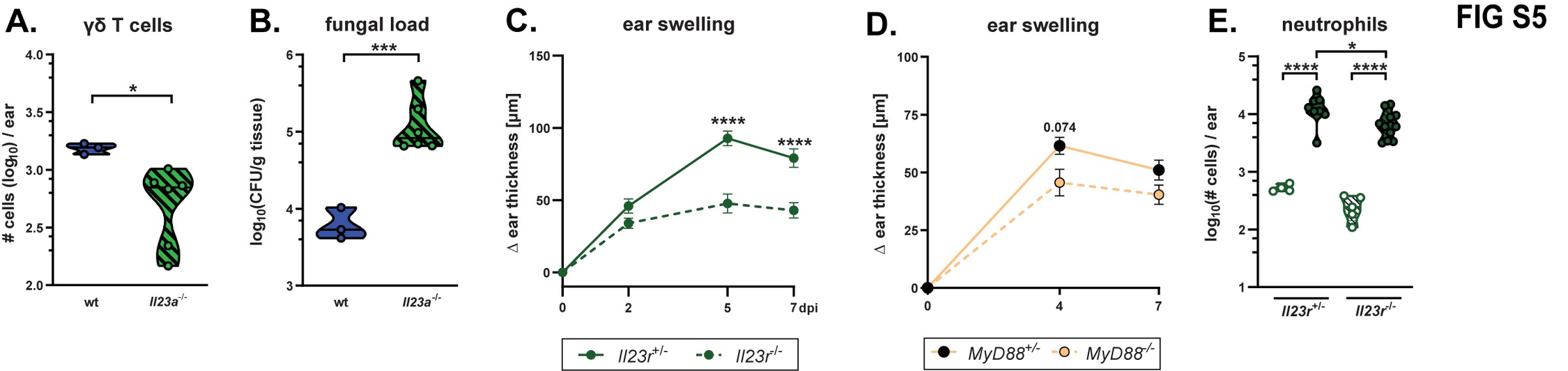

Supplement: S5 Fig — A.-B. The ear skin of Il23a-/- and C57BL/6 control mice was associated with M. pachydermatis for 7 days, when skin γδ T cells were quantified (A) and the fungal load (CFU) was assessed in the skin (B). C.-E. The ear skin of Il23r-/- and Il23r+/- littermate control (C, E) and of MyD88-/- and MyD88+/- littermate control mice (D) was associated with M. pachydermatis or vehicle treated for 7 days. Ear thickness kinetics (C-D) and skin neutrophils were quantified. Data are pooled from three (D) independent experiments with 2–5 mice per group each. The mean +/- SEM of each group is shown in C-D. In A-B and E, each symbol represents one mouse and the median of each group is indicated. Statistical significans was determined using unpaired Student’s t test (A-B) or two-way ANOVA (C-E). *p<0.05, ***p<0.001, ****p<0.0001. (TIF) [file ppat.1011668.s005.tif]

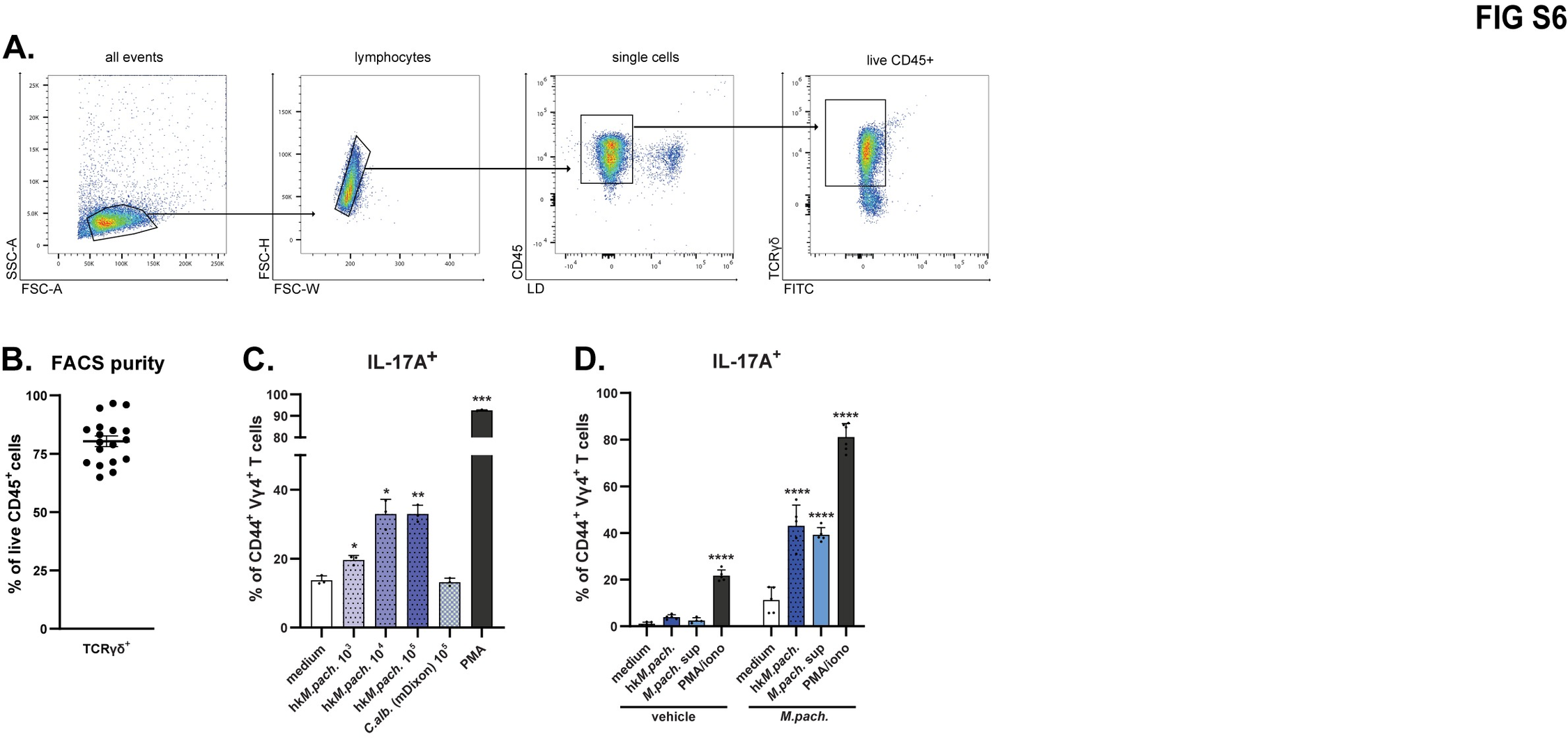

Supplement: S6 Fig — A.-D. γδ T cells were purified by FACS from dLN (A-C) or used as whole dLN suspension (D) of WT mice that were associated with M. pachydermatis (A-D) or treated with olive oil (vehicle, D) for 7 days and re-stimulated with the indicated compounds for 5 hours before quantification of IL-17A production by CD44+ Vγ4+ γδ T cells by flow cytometry as in Fig 7. Purity of FACS-sorted TCRγδ+ cells. shown by representative plots (A) and in a summary graph of data pooled from all experiments in Figs 7 and S5D (B). Re-stimulation with heat-killed M. pachydermatis (hkM.pach.) at different concentrations or with hk C. albicans grown in mDixon instead of YPD medium (C), or with hkM.pach. or M. pachydermatis supernatant (M.pach. sup; D). Stimulation with cell culture medium (medium) and PMA and ionomycin (PMA/iono) were included as reference for the response. In B, each symbol represents one mouse and mean+/-SEM is indicated. In C and D, each symbol represents one mouse, the mean+SD is indicated. Statistical significance between each stimulation condition and the respective medium control was determined using paired one-way ANOVA. *p<0.05, **p<0.01, ***p<0.001, ****p<0.0001. (TIF) [file ppat.1011668.s006.tif]
