# Supplementary material for: γδ T cells respond directly and selectively to the skin commensal yeast Malassezia for IL-17-dependent fungal control
Source: PLoS Pathog. 2024 Jan 12;20(1):e1011668. doi: 10.1371/journal.ppat.1011668 (PMC10810444; doi:10.1371/journal.ppat.1011668)
Supplement: Attachment — Submitted filename: cover letter&point-by-point reply_final.pdf [file ppat.1011668.s008.pdf]

University of Zurich[UZH]

Vetsuisse-Faculty
Section of Immunology

University of Zurich
Vetsuisse Faculty, Section of Immunology
Winterthurerstrasse 260
CH-8057 Zurich
Phone +41 44 635 88 01
www.immunology-vsf.uzh.ch

UZH, Vetsuisse-Faculty, Section of Immunology,
Winterthurerstrasse 260, CH-8057 Zurich

Prof. Dr. Salomé LeibundGut-Landmann
Associate Professor
Phone +41 44 635 87 21
Salome.leibundgut-landmann.uzh.ch

PLoS Pathogens
attn. Dr. Amariliz Rivera
Editor

Zurich, December 5th, 2023

RE: γδ T cells respond directly and selectively to the skin commensal yeast *Malassezia* for IL-17-dependent fungal control

Dear Dr. Rivera, dear Amariliz

We are pleased with the positive evaluation of our manuscript and the opportunity to revise the work for publication. We have followed the suggestions of the reviewers and generated new data to add more details on the antifungal response of γδ T cells in the murine skin. The results of these experiments have been incorporated in the revised version of the manuscript (new Figures 5D, new Supplementary Figures S1A, S3H, S4K-O; please note that due to the newly added figures, the number of some of the original figures has changed in the revised manuscript). We have also revised some figures panels (Figures 1C, 7D-F, S5E) and made modifications to the text, which are highlighted in red.

We have also made significant efforts to address the reviewers' concerns regarding the human data. Following the reviewers' suggestions, we performed new experiments that aimed at providing additional information regarding human γδ T cell subsets, activation, skin homing and cytokine production in response to *Malassezia*. While performing and analyzing those experiments, we noticed that the increase in HLA-DR and in the newly included skin homing marker CLA was not specific to γδ T cells, nor to any other T cell subset, but rather affected the entire PBMC population in a non-specific manner. Additional experiments then revealed that what we originally interpreted to be a *Malassezia*-induced shift in the activation marker HLA-DR was actually an artefact caused by the heat-killed fungal particles and suboptimal autofluorescence correction of the Cytek spectral analyzer. In consequence, we decided to remove the entire human dataset from the manuscript, in adherence with good ethical standards of scientific practice. We apologize for not having noticed the technical issue before submission of the original version of our manuscript.

We hope that you and the reviewers will comprehend our decision to remove the human data from the manuscript. While we do not exclude that human γδ T cells, especially those in the skin, may still respond to *Malassezia*, we currently have no means to further test this hypothesis experimentally due to lack of access to human skin samples.

ACCREDITED BY EAEVE/FVE
University of Bern | University of Zurich
vetsuisse-faculty

We further hope that you and the reviewers appreciate the strengths of the murine data of our study, which all three reviewers unanimously evaluated very positively during their initial review. We feel that the inclusion of additional experimental data on the murine γδ T cell response to *Malassezia*, which we added during the revision process, has further improved the quality of our manuscript. Attached we provide a detailed point-by-point response to each of the reviewer’s comments.

We appreciate that you give us the opportunity to have our work re-evaluated in this revised format. We are looking forward to your further consideration and decision regarding the paper’s suitability for publication in *PLoS Pathogens*.

We look forward to your reply.

Yours sincerely,

Salomé LeibundGut-Landmann

Point-by-point reply:

Reviewer 1 – Major Issues

The human experiments at the end of the manuscript read as very much an after-thought, allowing the authors to claim that the results also apply to humans. However, there are only very limited data and experiments included, which is a shame and problematic given the current claims that are made. If at all possible, this human aspect should be expanded**.**
a) Unlike the mouse experiments, some of which involved isolation of Vγ4 T cells, the human PBMC assay is quite a blunt tool to assess mechanism of Malassezia-mediated effects, as multiple immune subsets will be present, and therefore any γδ T cell activation may well be indirect and downstream of initial effects on other immune cells. Experiments purifying γδ T cells and incubating them with Malassezia would determine if the effects are direct or indirect.
b) The current data are quite limited: co-culture does not appear to result in significant differences in the % of live cells that are γδ T cells, and also it is unclear from the current experiments which subset of human γδ T cells express activation markers. From the percentages involved, it appears most likely to be the numerically dominant Vγ9Vδ2 T cell subset, but that could have been easily confirmed and is potentially relevant to understanding expression profiles of putative receptors for Malassezia components. This would be a priority to investigate.
c) In these human assays, induction of cytokine production by human γδ T cells is not addressed but is obviously of interest. Also, IL-17 production by human γδ T cells is very uncommon indeed, and it seems more likely the mechanisms of any human γδ response to Malassezia might therefore be very different.
d) Also, it is unclear if the activation induced by Malassezia (assessed here by HLA-DR expression) is restricted to γδ T cells or if other immune cells within PBMC are also activated (eg αβ T cells, NK cells). This could be easily addressed and would add to understanding of the PBMC assay.

Author reply: We would like to thank the reviewer for his/her valuable comments. We have undertaken several additional experiments to (i) subset the human γδ T cells and determine which of the subsets present in peripheral blood would be responsible for the observed signal in response to *Malassezia* spp.; (ii) determine the cytokines produced by the responding γδ T cells; (iii) investigate whether the response to *Malassezia* spp. was restricted to γδ T cells or also observed in TCRγδ-negative T cell subsets; and (iv) complement the activation readout, which was limited to HLA-DR, with the skin-homing marker CLA. While performing and analysing these experiments, it occurred to us that the shift in HLA-DR and CLA was not specific to γδ T cells, nor any of the other T cell subset, but rather observed in the entire PBMC population. Additional experiments then revealed that what we originally interpreted to be a *Malassezia*-induced shift in the activation marker HLA-DR was actually an artefact caused by the heat-killed fungal particles and suboptimal autofluorescence correction of the Cytek spectral analyzer. We provide below an extract of our extensive analyses that led to this conclusion.

At the current stage, we are unable to confirm activation of circulating γδ T cells by *Malassezia* and therefore decided to remove all human data from the manuscript. While we speculate that the fungus may act on human γδ T cells in skin, we currently have no access to human samples, which would allow us to generate experimental evidence for this hypothesis. Extending our study into this direction is planned for the future but goes beyond the scope of the present manuscript.

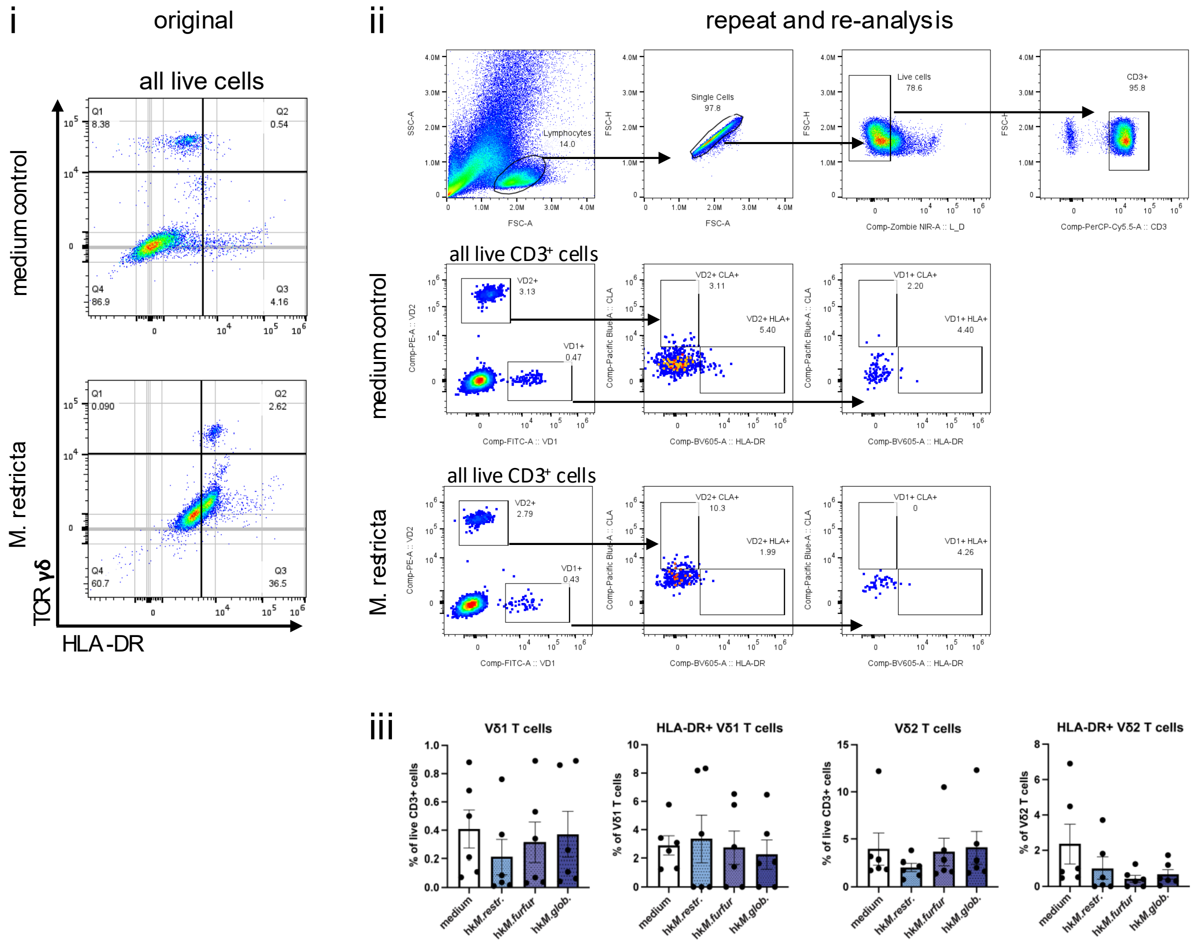

*Figure A: Human PBMCs were cultured for three days in the presence of heat-killed Malassezia spp. or control medium and analysed by flow cytometry. (i) Reanalysis of original data revealing a shift in HLA-DR of the entire PBMC population in M. restricta-*stimulated vs. medium control *condition. (ii) Optimized gating of newly generated data including Vδ1 and Vδ2 subsets as well as HLA-DR and CLA expression. (iii) Percentages of Vδ1 and Vδ2 T cells of total live CD3⁺ cells and of HLA-DR⁺ proportions within Vδ1 and Vδ2 T cell populations. Data shown are from two independent experiments. Each symbol represents one donor, the mean+SEM is indicated.*

Reviewer 1 – Minor Issues

(i) The study purports to be relevant to human infection, but the Introduction is exclusively focussed on mice, with essentially no references to human γδ T cells. Some comments are incorrect when applied to the human γδ T cell compartment, such as in relation to innate pre-programming, which is the case for Vγ9Vδ2 T cells, but not for Vδ2neg T cells. If aiming to be relevant to the human compartment, then references to cover both innate-like Vγ9Vδ2 T cells, and adaptive-like Vd2neg and Vγ9negVd2 T cells, should be included. Some relevant references would be those by the Willcox group, chiefly Davey et al, 2017 (PMID 28248310), Davey et al, 2018a and 2018b (PMID 29720665 and 29680462); and Willcox CR et al, 2020 (PMID 33084045). If not aiming to apply to human γδ T cells, then the introduction text should make it clear the authors are talking exclusively about the mouse γδ T cell compartment. Given the inclusion of human data later in the manuscript it would seem logical to broaden the scope to cover human γδ T cells.

Author reply: Please see our reply to reviewer 1's major comment above. Because we had to remove all human data from the manuscript, we have refrained from expanding the introduction towards

human γδ T cells. We have however adjusted the text to make clear which statements apply specifically to mice and not to humans (lines 64ff).

(ii) Given the weakness of the human dataset (see comments in Part II), elements of the results appear to be over-interpreted.
- In the Abstract, it is hard to justify the comment regarding 'confirmed the relevance of this fungus-specific response' given the dearth of information about it in the human setting (eg which cytokines are produced (most likely not IL-17), whether there is direct/indirect activation of γδ T cells). This is compounded by the fact that Malassezia is a skin fungus and yet these assays are performed on PBMC, so it's not exactly a physiological setting – something I feel should be included as a caveat in the Discussion.

- In the Discussion, the current text states that 'the relevance of this Malassezia-derived moiety as a specific agonist for γδ T cells is further underlined by the ability to activate human peripheral blood γδ T cells'. This is a major stretch, as just because there is an effect on human γδ T cells does not mean the mechanism or components are the same. It is therefore entirely unclear from the current limited dataset if the mechanism is conserved or not. Given the general lack of alignment between mouse and human γδ T cells, a similar mechanism would arguably be more surprising and certainly has not been proven. Of relevance, I don't believe the involvement of a soluble factor from Malassezia has been shown in the human system – the manuscript only refers to use of heat-killed Malassezia in the PBMC assay. Furthermore, as stated above, a specific effect on γδ T cells (as opposed to an indirect effect, and/or an effect on both γδ T cells and other cells) has definitely also not been proven. So discussion of the human results as denoting a Malassezia-derived moiety that is a specific agonist for γδ T cells is premature.

Author reply: Because we removed all human data from the manuscript we have also removed all statements referring to these data from the text.

(iii) A final query for the Authors is whether they can exclude the possibility that the ability of Malassezia-exposed Vγ4 T cells to sense a soluble Malassezia-derived component is TCR-dependent. Could it be that the initial stages of the Vγ4 response are cytokine driven (IL-23, IL-1) and TCR-independent, but the post-exposure, direct sensing of the soluble Malassezia component is influenced by changes in the TCR repertoire after initial exposure. A relevant experiment (whether practical or affordable) might be to assess the Vγ4 subset repertoire before and some time after 1st exposure to Malassezia. Major clonotypic focussing might imply antigen-specific selection and shaping of the response by exposure to the fungus. If this possibility can't be excluded, this is something that could be highlighted in the Discussion.

Author reply: Thank you for raising this interesting hypothesis. Our results suggest indeed that the initial stages of the Vγ4 response are cytokine driven (IL-23, IL-1), while fungal metabolites are sufficient post-initial exposure to elicit IL-17 production. To assess whether TCR is involved in direct sensing of *Malassezia* metabolites we made use of Nur77 reporter T cells. We found no significant activation of the TCR in γδ T cells from Nur77 reporter mice after re-stimulation with *Malassezia* (Figure 5E-H) and therefore stated in the discussion "*Malassezia* stimulation of γδ T cells does not substantially engage the TCR" (line 427-428). Additional experiments are warranted to examine possible shifts in the repertoire in response to fungal exposure and to address the clonality of the γδ T cells, which however exceed the scope of this study.

(iii) A final point is that on occasion figure panels are called out in the text out of order, and I believe Figure 8F is not called out in the text at all.

Author reply: Thank you for noticing. We have corrected this oversight (line 365).

Reviewer 2 – Minor Issues

1. Page 8 Ln 235 spelling, trangene positive

Author reply: Thank you for spotting this spelling error, which we have now corrected (line 239).

Reviewer 3 – Major Issues

1) One concern for this manuscript is assigning a direct role to the IL-17 gd T cells in providing protection from M.pach infection. Specifically, an example of this is the FTY720 experiments. FTY720 blocks egress of all lymphocytes from the lymphoid tissue, including the CD4 TH17 cells. How can the role of the CD4 TH17 cells be differentiated from the IL-17 gd T cells in protection from M.pach infection in the FTY720 experiments if both are blocked from leaving the dLN? The authors show that there is a higher number of gd T cells in the dLN at 44 days, but this does not rule out the TH17 cells from being able to expand that are in the dLN and aid in the protective responses.
An adoptive transfer of the primary infected gd T cells into a naïve mouse could allow the authors to determine if the gd T cells are sufficient to provide protection from M.pach infection and could help in differentiating their role compared to TH17 cells.

Author reply: Thank you for raising this point. We agree that the FTY720 experiment does not exclude a potential contribution of Th17 cells to fungal control and have highlighted this in the text (lines 217ff).

We have refrained from performing an adoptive transfer as we believe that the significance of such an experiment would be limited due to the challenge of defining appropriate controls. Adoptive transfer of γδ T cells from an *Malassezia*-infected into a naïve mouse will not conclusively inform whether γδ T cells are superior to αβ T cells in limiting fungal colonization. We speculate that adoptive transfer of αβ T cells from a *Malassezia*-infected mouse into a naïve mouse, in which γδ T cells have not previously been activated, would likely also aid fungal control if they are the sole population of IL-17 producers.

Instead, we performed an experiment to deplete CD4+ T cells (including Th17 cells) by means of a depleting antibody prior and during colonization. This did not affect the fungal load in the skin. However, while depletion was highly efficient in blood and lymph nodes, the antibody was only partially effective in the skin, limiting the interpretability of the experiment. We therefore did not include the data in the manuscript but provide the results to the reviewer (see Figure below).

Together, we would like to emphasize that our study does provide ample evidence for γδ T cells representing the dominant source of IL-17 in the *Malassezia*-associated skin and that they are critical for fungal control over a prolonged period of time.

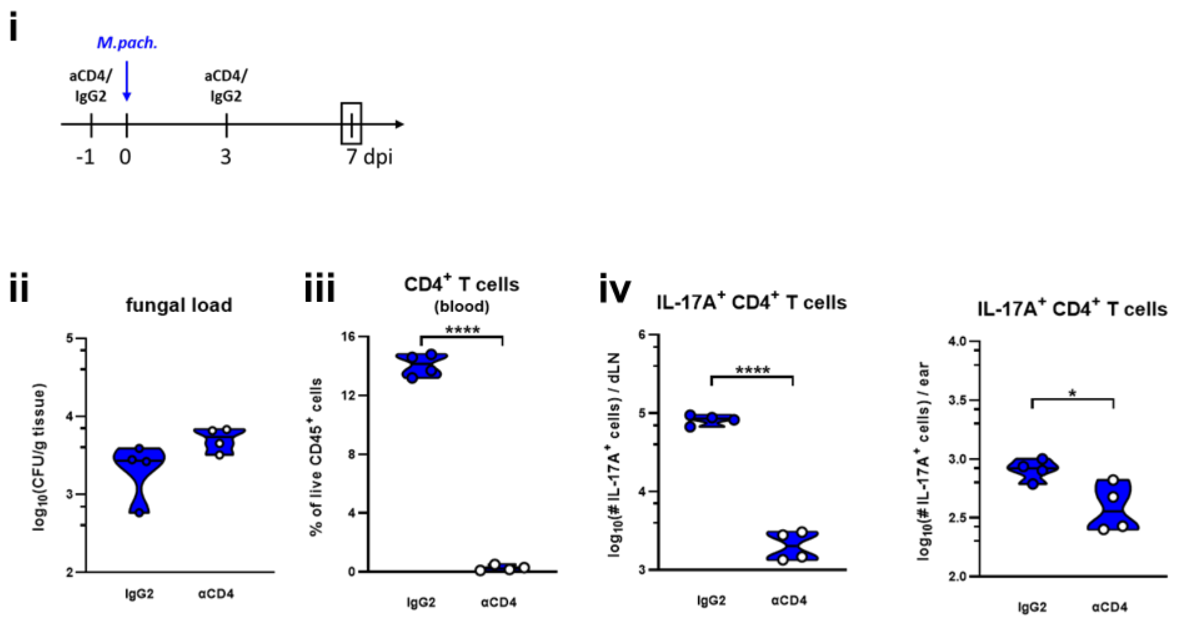

***Figure B**: C57Bl/6 wildtype mice were injected intraperitoneally with an anti-CD4 depleting antibody (clone GK1.5 (BioXCell), 500 µg / animal) or rat IgG2b isotype control (BioXCell) control 1 day prior and again 3 days after fungal association (i). Seven days after fungal association, we assessed the skin fungal load (ii), the frequency of CD90+CD4+ T cells among CD45+ cells in the blood (iii), and the number IL-17+ CD4+ T cells in the draining lymph nodes and the skin after ex vivo staining with a non-competing anti-CD4 antibody (clone RMA4.4) (iv).*

Also, line 195 states that IL-17 production was increased in gd Tcells in response to fungal re-exposure (4D), but does not mention that a very similar increase in IL-17 was also found in the CD4+ cells (FigS3E). Please add a description of this result into the main text.

Author reply: Thank you for pointing this out. We have adjusted the text in lines 198 to point out the increase in CD4+ cells in the dLN.

2) Why are ear thickness/inflammation only shown for some knockout mice and not for others? For example, the FTY720-blockade experiment (Fig4), did this blockade reduce the inflammation as found in the Tcdr-/- and IL-17af-/-? What happened to the ear thickness of the CD11c DTR mice (Fig 5)? Same for figures 6 and S4. Was the inflammation of the ear the same between the control and knockout mice? If the inflammation is tied to the actions of the IL-17 gd Tcells (which are unaltered in most of these mice), then the thickness should not be affected. If it is, that should be discussed. These data are important for interpreting how inflammation is related to the level of IL-17 gd Tcells.

Author reply: Yes, indeed we observed a link between γδT17 cells and inflammation. While complete absence of γδ T cells (*Tcrd-/-*) or IL-17 (*Il17af-/-*) resulted in reduced ear swelling, a partial reduction in skin IL-17 γδ T cells as in FTY720-treated mice did not alter ear swelling. Furthermore, in CD11c-DTR, *Card9-/-*, *Clec7a-/-*, *Clec4n-/-*, *Clec4e-/-*, *Tlr23479-/-* mice, in which γδ T cells were not reduced, ear swelling was still as pronounced as in the respective controls at 7 dpi. We have added these data in the new figures panels Fig. 5D, Fig S3H, Fig S4K-O, and adjusted the text accordingly (lines 215, 242, 268, 282).

3) Some of the experiments, specifically ones involving IL23R+/- mice (Fig 8D,E), only have 2 animals in some of the groups. These should be repeated to reach a sufficient power for interpretation. Additionally, the figure legend says that data are pooled from 2 experiments with n=2-5 mice per group. Were the vehicle controls only done once with 2 mice?

Author reply: We have increased the number of animals in all groups and corrected the mistake in the corresponding figure legend. See Fig 7D-F and S5C,E.

Reviewer 3 – Minor Issues

1) The neutrophils numbers of the IL-17af-/- mice reach the level of WT mice ~day7. It's interesting that at that point the fungal load in those mice then begins to drop, but they never clear the infection. It would be beneficial to discuss why this may be. Do the neutrophil levels drop again. Is IL-17 important for their retention and/or activity at this stage of infection?

Author reply: The persistence of a low fungal load in IL-17af-/- mice is indeed intriguing. We observed similar colonization kinetics after oral colonization of IL-17-deficient mice with Candida albicans (PMID 32719409). We have not investigated neutrophil dynamics and function or their IL-17-dependency beyond day 7 and can therefore not answer this question. We have however extended the text in lines 126ff speculating about the underlying cause of the observed IL-17-independent dynamics of the fungal load.

2) gd Tcells are also known for being able to produce IFNgamma (as opposed to IL-17) and in some circumstances have been found to co-produce both IL-17 and IFNgamma in response to microbes. Because the IFNgamma producing gd Tcells are known to be more protective, it may be important to know whether IFNgamma may also be affected or even produced by the gd T cells in response to M.pach. It would be beneficial to add this to the discussion.

Author reply: We show in Figure 2K that *Malassezia*-responsive γδ T cells do not produce detectable levels of IFN-γ. We have now further highlighted this finding in the discussion (line 395ff).

3) Your data shows that gd T cell deficient mice (Fig3) have a stronger initial recruitment of neutrophils and are able to eventually clear the M.pach infection compared to IL17af-/- mice (Fig1). This supports the involvement of a separate cell(s) in the Tcrd-/- mice that are responding to IL-17 to eventually clear M.pach skin infection. A discussion on this would be beneficial.

Author reply: Thank you for raising this point. We have highlighted this in the original version of our manuscript (now: in line 175ff) and have now added a sentence in the discussion to increase the emphasis (lines 395ff).

4) In Fig1C, the tissue inflammation of the IL-17af-/- mice appears to go up visually from day 2 to day 4, whereas in the graph of the combined data (1B) it is shown to slightly drop or stay the same from day 2 to 4. And visually, there isn't a big difference between day 4 and 7 in the images, but there is in the graph of the measured thickness. Are the images presented in 1C for the IL-17af-/- mice truly representative of the overall result depicted in Fig1B?

Author reply: Thank you for pointing this out. There was indeed a mix-up of the images of day 4 and vehicle skin of IL-17af-/- mice in Figure 1C. The error has now been corrected.

5) Human and mouse gd T cells are known to be different. Did you characterize which human gd Tcells were expanding in response to Malassezia? Did they induce IL-17? These experiments could add a lot of support towards a similar mechanism across hosts if included.

Author reply: Please see our detailed answer to reviewer 1's major comment above regarding the data on human γδ T cells.

6) It is mentioned in the text that neutrophils are infiltrating into the skin as observed by histology (line 119). Please provide a higher magnification image and labelling to support this statement or remove the "and by histology" statement as there is already flow cytometry data demonstrating this.
Author reply: To support our statement, we have added an image at higher magnification in Fig S1A.

7) Why was PBS used as a control for anti-Gr1 instead of an isotype antibody (Fig 7G-K)?

Author reply: Indeed, a control with isotype would have been better, but unfortunately, we only included injection controls with PBS. In similar depletion experiments performed earlier in our lab, we did not observe any effect of isotype versus PBS treatment with regards to our readouts.
